# Pattern Formation in Populations with Density-Dependent Movement and Two Interaction Scales

**DOI:** 10.1371/journal.pone.0132261

**Published:** 2015-07-06

**Authors:** Ricardo Martínez-García, Clara Murgui, Emilio Hernández-García, Cristóbal López

**Affiliations:** 1 IFISC (Instituto de Física Interdisciplinar y Sistemas Complejos), CSIC-UIB, Campus UIB, Palma de Mallorca, Spain; 2 Department of Ecology and Evolutionary Biology, Princeton University, Princeton NJ, 08544-1003, United States of America; Shanxi University, CHINA

## Abstract

We study the spatial patterns formed by a system of interacting particles where the mobility of any individual is determined by the population crowding at two different spatial scales. In this way we model the behavior of some biological organisms (like mussels) that tend to cluster at short ranges as a defensive strategy, and strongly disperse if there is a high population pressure at large ranges for optimizing foraging. We perform stochastic simulations of a particle-level model of the system, and derive and analyze a continuous density description (a nonlinear diffusion equation). In both cases we show that this interplay of scale-dependent-behaviors gives rise to a rich formation of spatial patterns ranging from labyrinths to periodic cluster arrangements. In most cases these clusters have the very peculiar appearance of ring-like structures, i.e., organisms arranging in the perimeter of the clusters, which we discuss in detail.

## Introduction

Individual based models are of great relevance in many disciplines, ranging from condensed matter [1] to biology [2, 3], economics and social dynamics [4]. They allow to simulate some simple dynamical rules and study its consequences at a global population scale. In an ecological context individual based models have gained in importance [5–7], and are commonly used to investigate collective behavior and the emergence of patterns, which are central issues in theoretical ecology [8].

In this paper, we propose a model to study the formation of spatial patterns in a population of organisms in which interactions affect their mobility. We assume that, during the time scales of interest here, no other dynamical processes such as birth, reproduction and death occur. The movement of any individual depends on the distribution of its conspecifics at two length scales. We thus focus on the problem of group formation and spatial aggregation [9–12], although this approach may be used in the more general context of collective movement [13] (including birds flocks [14, 15], fish swarms [16, 17], and mammals herds [18]), and also to address the effect of spatial degrees of freedom in evolutionary problems [7].

Spatial aggregation is a widespread phenomenon in living systems, resulting from the combination of individual movement with interspecific and intraspecific interactions [3, 19]. Therefore, a mathematical description of group formation should include all these mechanisms, and several ways of integrating collective interactions with individual movement have been proposed [2, 13, 20–23]. A very important type of models considers that interactions influence only the movement of the individuals disregarding any other intra- and inter-specific interactions. They are relevant to study animal or organism dispersal wherever there is an increase of the diffusivity with the local density because of population pressure [2, 3]. Extensions of these works also account for the effect of conspecifics located at separated positions [2, 20, 24], including nonlocal spatial interactions. This family of models results in nonlocal nonlinear diffusion equations [25, 26] for the population density. From a biological point of view, they usually account for a single class of interactions, and diffusivity depends on the population density over one neighborhood of the focal individual. However, in a more general framework, many different interactions of diverse nature are relevant within a population, so these single-scale approaches might not describe the complete set of processes taking place. For instance, high long-range densities (i.e. densities of others within a long distance around a focal one) may encourage animal mobility due to intraspecific competition for resources, while on a shorter spatial scale individuals may arrange in cooperative aggregations so that the predation risk decreases. Also in the decision-making process that underlies collective movement, animals choose how to move depending on their neighbors at different distances, so they guarantee the cohesion of the group [14, 27, 28]. In a rather different context, the formation of patterns of vegetation has been also traditionally thought to be a consequence of the interplay between plant interactions at two different scales: short-range facilitation and long-range competition [29–33], although this has been a contentious claim [34]).

Mussel beds are one of the paradigmatic examples of spatial aggregation in nature. Experimental works have shown that the origin of the aggregates lies in the interaction among individuals [35], although modified by the interplay between the whole population and the environment [36]. Many theoretical attempts have proposed mathematical models to unveil the mechanisms that, acting at different spatial scales, stabilize the patterns. Two families of models have arisen, both of them containing competition for resources on a large scale and facilitation (aggregation promotion to diminish wave stress and predation risk) on a short scale: a) studies considering the dynamics of two populations (the algae and the mussels) [37–39]; and b), an study that focuses only on the dynamics of the population of mussels (unique species model), including the interaction with the environment (i.e, algae) in nonlocal spatial terms [40].

Within this framework, but mainly motivated by [40], we present a model of interacting particles where the mobility of the individuals, i.e. its diffusivity, depends on two spatial scales. Movement is encouraged when the density is high in a long-range (competition), and inhibited if it is so in a short-range (i.e., cooperative aggregations are favored at shorter scales). The principal novelty of our work with respect to [40] is the Brownian nature of the motion of the particles in the discrete description of the system and its generality, that allows the exploration of different relationships between the diffusivity and the density of individuals. We will perform a numerical study of this stochastic picture and compare the results with the equivalent deterministic population level approach.

In the following sections pattern formation will be studied combining numerical and analytical techniques both in the discrete-particle dynamics and its continuous-field density equation.

## Materials and Methods

### Individual-based dynamics

Let us consider a population of *N* individuals undergoing Brownian movement with a diffusion coefficient that depends on the densities of conspecifics at two separated length-scales: a mean density ${\tilde{\rho }}_{s}$ at short range, *R*_*s*_, and a mean density ${\tilde{\rho }}_{l}$ over a long one, *R*_*l*_ (*R*_*s*_ < *R*_*l*_). We will denote the position of each particle by **r**_*i*_ = (*x_i_*, *y_i_*) at any time *t* in a two-dimensional square system of lateral extent *L* with periodic boundary conditions.

**Fig 1:**
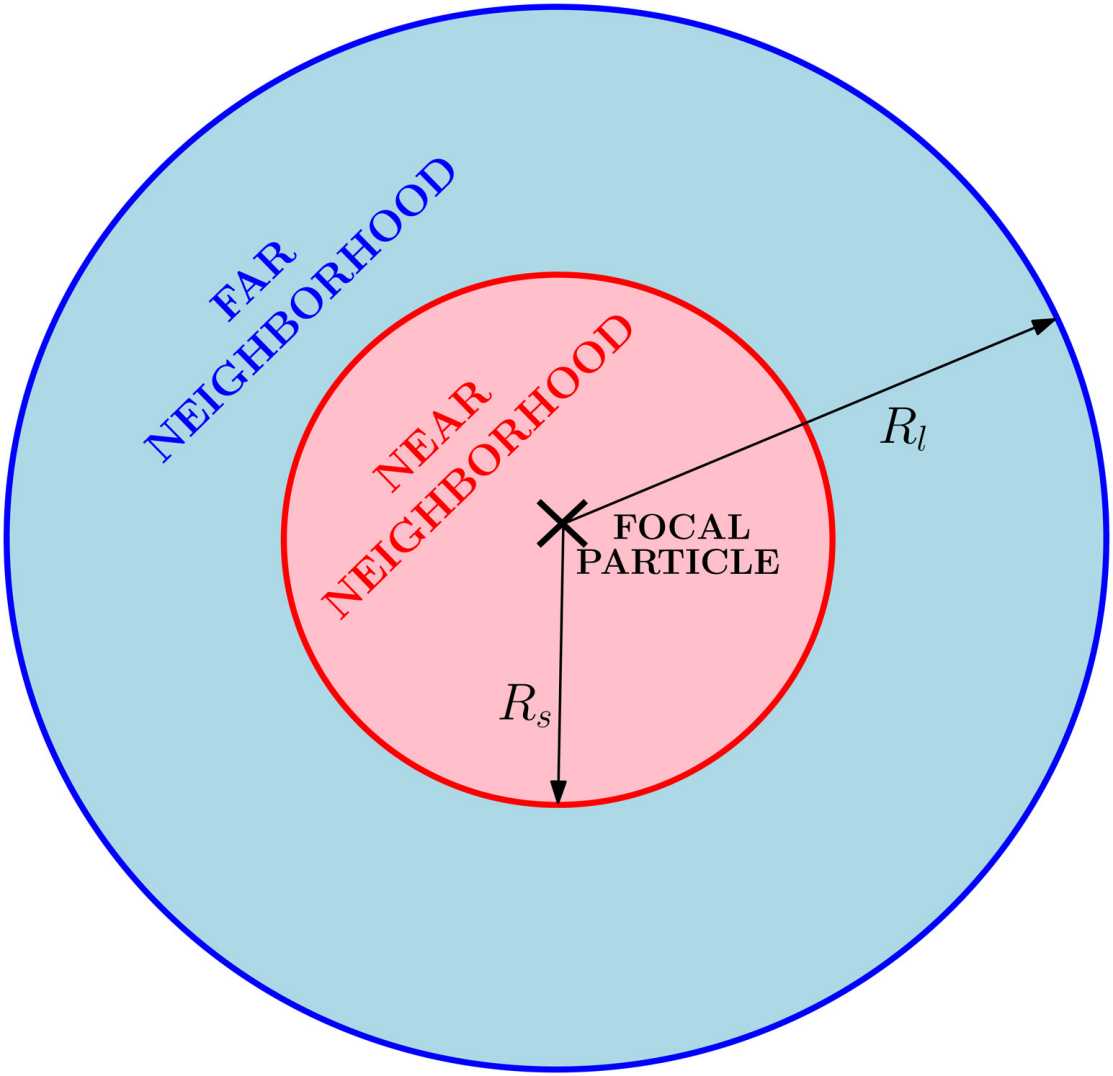
Interaction neighborhoods. Short- and long-range interaction neighborhoods for a given individual. The regions are defined by their radius *R*_*s*_ and *R*_*l*_ respectively.

The dynamics of each particle *i* = 1, … *N* is then given by

$${\dot{r}}_{i}=\sqrt{2D(r_{i},{\tilde{\rho }}_{s},{\tilde{\rho }}_{l})}\eta_{i}\left(t\right),$$ (1)

where the diffusivity *D* is, in general, a positive continuous function of ${\tilde{\rho }}_{l}$ and ${\tilde{\rho }}_{s}$. ***η***_i_(t) is a white Gaussian vector noise with zero mean and with time-correlation matrix given by 〈***η***_*i*_(*t*)***η***_*j*_(t′)〉 = **1** *δ*_*ij*_ *δ*(*t* − *t*′). **1** is the identity matrix. Eq (1) should be interpreted within the Itô calculus, since the stop/movement behavior is assumed to occur at the beginning of each time step [25]. The mean densities are defined as:

$${\tilde{\rho }}_{\mu }\left(r\right)=\frac{N_{\mu }}{\pi R_{\mu }^{2}},$$ (2)

with *μ* ≡ *s*, *l*. *N*_*s*_ and *N*_*l*_ are the number of individuals found in a near and far neighborhood of the particle at **r**, respectively (see Fig 1). Note that, since the number of individuals does not change, the global density *ρ*_0_ = *N*/*L* ^2^ remains constant in time.

The main focus of this approach is on species that switch between the tendency to aggregation and to dispersion as the number of surrounding individuals increases at different length scales. In particular, we address the case of competitive long range interactions and facilitation at a shorter scale. This is the observed framework in mussel beds, where patterns appear due to the interaction between two opposing mechanisms: competition for resources at a large scale and defense against predators and waves stress at shorter distance [36, 37, 40]

To model this behavior we consider that the diffusivity, *D*, is enhanced with increasing the long-range density, and reduced with increasing short-range density. This can be written as $D=D_{0}g(a-b{\tilde{\rho }}_{s}+c{\tilde{\rho }}_{l})$ if *g* is an arbitrary function with positive derivative, ∂_*x*_ *g*(*x*) > 0. *D*_0_ is a constant diffusivity and *a*, *b*, and *c* are positive parameters. Note that with the expression $a-b{\tilde{\rho }}_{s}+c{\tilde{\rho }}_{l}$ we indicate, as mentioned before, that the *tendency* of a particle to move decreases with the short-range mean density ($-b{\tilde{\rho }}_{s}$) and increases with the long-range one ($c{\tilde{\rho }}_{l}$). The function *g* takes its maximum (minimum) value in the limit ${\tilde{\rho }}_{l}\gg {\tilde{\rho }}_{s}$ (${\tilde{\rho }}_{s}\gg {\tilde{\rho }}_{l}$). For simplicity we restrict to the case 0 ≤ *g* ≤ 1 so the diffusivity of the particles varies between 0 and *D*_0_. *D*_0_ is the diffusion coefficient of the population when movement is extremely promoted (${\tilde{\rho }}_{l}\gg {\tilde{\rho }}_{s}$).

With the above mentioned properties of *g* we choose as an example (the main results are independent of the particular *g*)

$$g\left(r_{i}\left(t\right)\right)=\frac{1+\tanh[2(a-b{\tilde{\rho }}_{s}\left(r_{i}\right)+c{\tilde{\rho }}_{l}\left(r_{i}\right))-1]}{2},$$ (3)

where parameters *b* and *c* weight the importance of the short and the long-range densities, respectively, and parameter *a* gives the diffusivity of an individual when short and long-range densities are equal and have the same weight. Notice once again that *g* → 0 if ${\tilde{\rho }}_{s}\gg {\tilde{\rho }}_{l}$ and *g* → 1 if ${\tilde{\rho }}_{l}\gg {\tilde{\rho }}_{s}$.

### Continuum description

The particle dynamics given by Eq (1) allows an intensive numerical exploration. To complement the study and obtain analytical results it is essential to have a simplified continuum equation of the model, where the population is described in terms of a collective variable: the local density of individuals *ρ*(**r**). This equation can be derived following Dean’s approach [41] from the stochastic particle dynamics presented in the previous section, which uses Itô calculus. Considering a mean-field approximation (i.e., neglecting fluctuations in the density) we obtain for the mean particle density

$$\frac{\partial \rho (r,t)}{\partial t}=D_{0}\nabla^{2}[g\left({\tilde{\rho }}_{s},{\tilde{\rho }}_{l}\right)\rho \left(r,t\right)],$$ (4)

where the mean long- and short-range densities are computed as

$${\tilde{\rho }}_{\mu }\left(r,t\right)=\int G_{\mu }\left(r-r^{\prime }\right)\rho \left(r^{\prime },t\right)dr^{\prime },$$ (5)

where *G*_*μ*_, with *μ* ≡ *s* or *μ* ≡ *l*, are the short and long range kernel functions that define both interaction scales. The kernel functions are normalized and have units of inverse of area

$$\begin{array}{c} G_{\mu }\left(|r|\right)=\{\begin{array}{ccc} \frac{1}{\pi R_{\mu }^{2}} & \text{if} & \left|r\right|\leq R_{\mu }\\ 0 & \text{if} & |r|>R_{\mu }, \end{array} \end{array}$$ (6)

*R*_*μ*_ (*μ* ≡ *s*, *r*) define, as in the individual based approach, the short and long interaction ranges, respectively.

## Results

**Fig 2:**
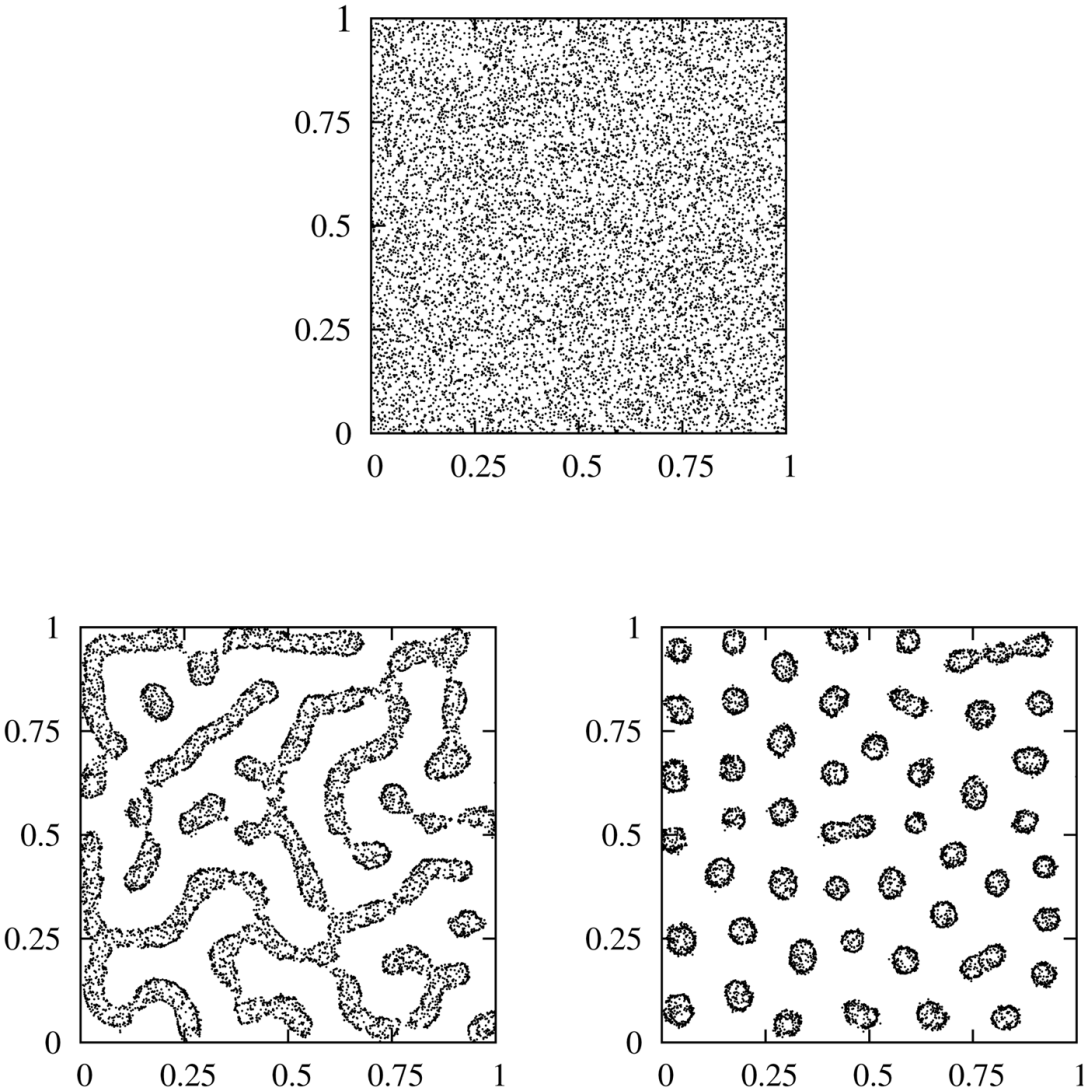
Spatial distribution of the population from the particle-level model. Spatial distribution at long times of a population of 10^4^ individuals using the dynamics of Eq (1) with a short interaction range *R*_*s*_ = 0.05 and a long interaction length *R*_*l*_ = 0.1. *D*_0_ = 10^−4^, *a* = 1 in all the panels. Every individual is plotted as a small black dot. The system is a square area of lateral size *L* = 1 with periodic boundary conditions. Upper panel: *b* = 3.5 × 10^−4^, *c* = 7.0 × 10^−4^ (homogeneous distribution). Left bottom panel: *b* = 8.5 × 10^−4^, *c* = 7 × 10^−4^ (labyrinth pattern). Right bottom panel: *b* = 4.3 × 10^−4^, *c* = 3.9 × 10^−4^ (spot pattern). Note the rings with higher density in the border.

A direct exploration of pattern formation in the model starts from Monte Carlo numerical simulations of the individual-based dynamics given by Eq 1. To unveil the relationships between the two spatial scales that promote the formation of spatial structures, we isolate in our analysis the relative importance of the short and long-range densities fixing all the parameters of the model (*R*_*s*_, *R*_*l*_, *D*_0_, *N*, *a*; see caption of Fig 2 for details), except *b* and *c*, that weight the influence of ${\tilde{\rho }}_{s}$ and ${\tilde{\rho }}_{l}$ on the diffusivity.

Depending on the relationships between this pair of parameters the population may show a homogeneous distribution (Fig 2, top panel) or arrange developing spatial aggregations (bottom panels of Fig 2). Two classes of patterns are observed: labyrinth distributions and isolated spots [35, 40] arranged in a hexagonal matrix. A relevant and singular feature is the shape of the aggregations, with most of the individuals clumped in the borders of the cluster and an almost empty inner area. Similar ring-like structures have been previously reported in plant ecology and studied with models based on mechanisms very different form ours, but that share with our approach the presence of competitive and facilitative interactions [42, 43].

**Fig 3:**
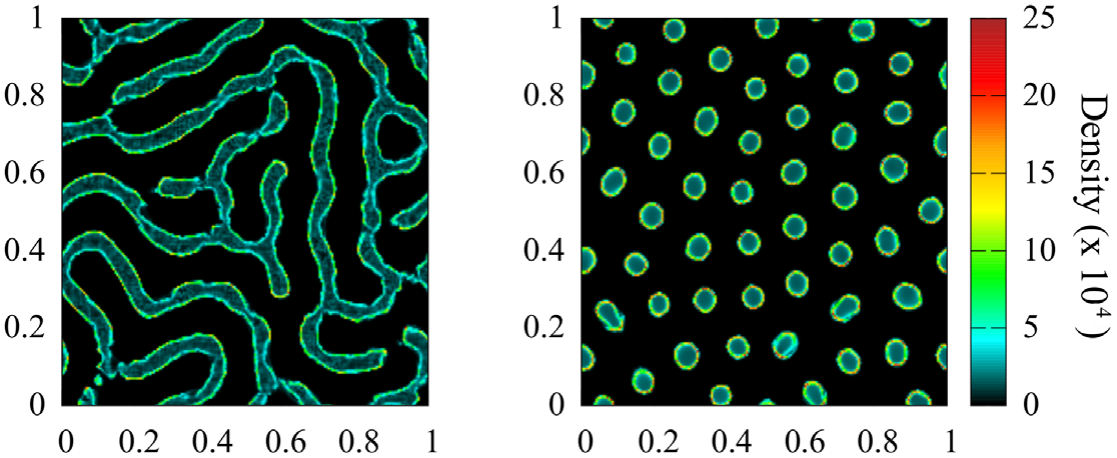
Solutions of the continuous density equation. Long time solution of Eq (4) with a short interaction range *R*_*s*_ = 0.05 and a long interaction length *R*_*l*_ = 0.1. *D*_0_ = 10^−4^, *a* = 1 and density *ρ*_0_ = 10^4^ in all the panels. An Euler algorithm was implemented and integration performed over a square area with lateral size *L* = 1 and periodic boundary conditions. Left panel: *b* = 8.5 × 10^−4^, *c* = 7 × 10^−4^ (labyrinth pattern). Right panel: *b* = 4.3 × 10^−4^, *c* = 3.9 × 10^−4^ (spot pattern).

**Fig 4:**
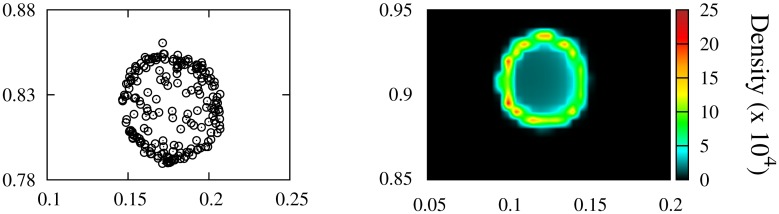
Comparison of a single ring-like structure in both approaches. Detailed distribution of the individuals within one of the groups of the spotted pattern in the discrete model (Left) and of the density in one of the patches in the solution of the continuous equation (Right). Parameters: *b* = 4.3 × 10^−4^, *c* = 3.9 × 10^−4^, *D*_0_ = 10^−4^, *a* = 1, *R*_*l*_ = 0.1 and *R*_*s*_ = 0.05 in all the panels.

A deeper understanding of the pattern formation dynamics can be addressed using the continuum description given by Eq (4). To corroborate the correspondence between the individual based description by Eq (1) and the continuous approach in terms of Eq (4), we numerically integrate Eq (4). Kernels are fixed as given by Eq (6) and the parameters take the same values as in Fig 2 to allow a direct comparison with the discrete simulations (see caption of Fig 3 for details). The laberynth and spot patterns showed in Fig 3 exhibit a good agreement with the distributions of Fig 2 resulting from the stochastic particle dynamics. In particular, details of hollow clusters for both micro and macro descriptions are plotted in Fig 4. The distribution of the particles within the clusters is a particularly interesting question that will be discussed later in this section.

**Fig 5:**
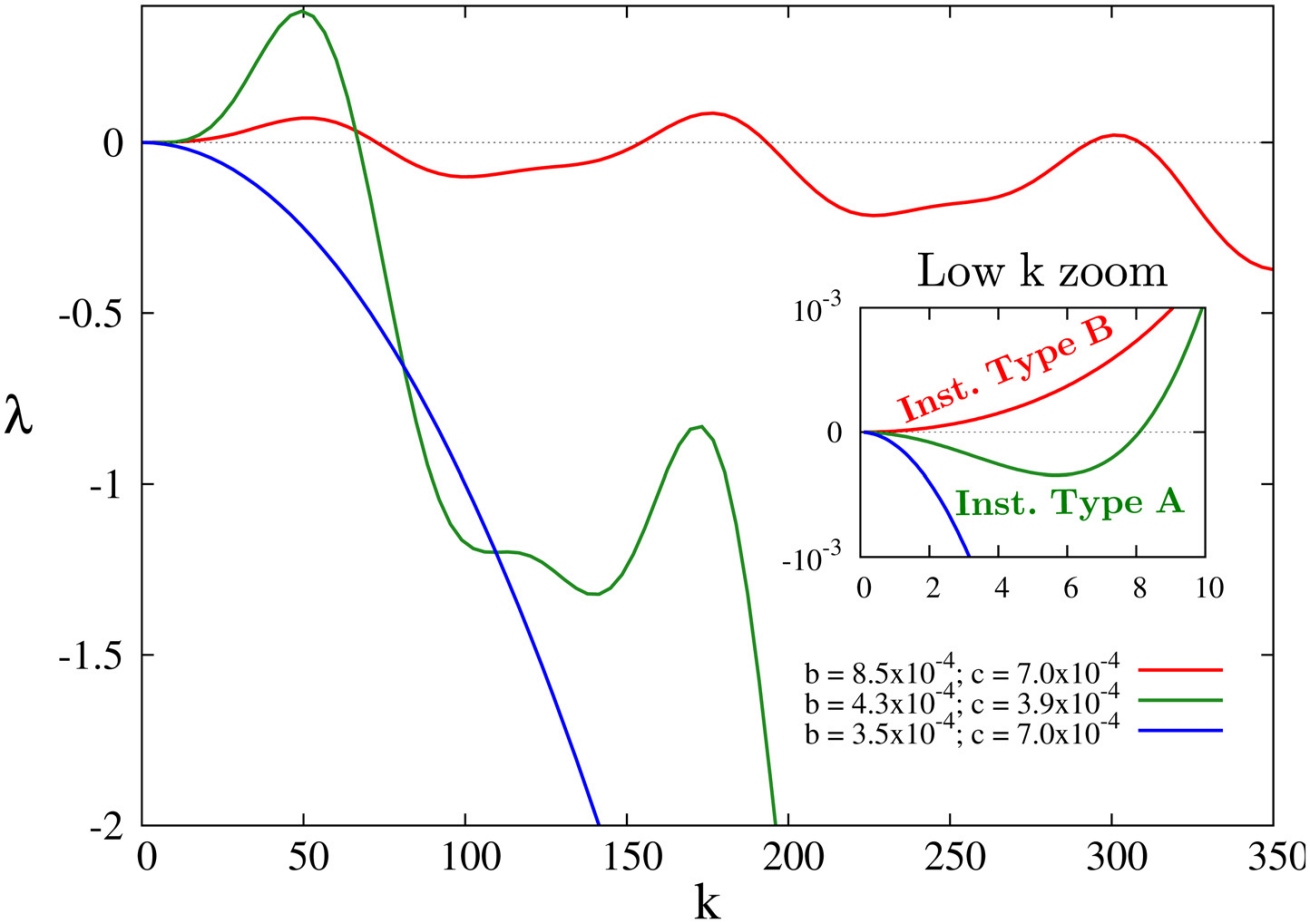
Perturbation growth rate. Perturbation growth rate as a function of the wavenumber, Eq (8), for different values of the parameters *b* and *c*. *R*_*s*_ = 0.05, *R*_*l*_ = 0.1, *D*_0_ = 10^−4^ and *a* = 1.

We continue with the analytical approach performing a linear stability analysis of Eq (4). We note that the homogeneous distribution of the *N* individuals in the box of size *L*, i.e. *ρ*(**r**, *t*) = *ρ*_0_ = *N*/*L* ^2^ always provides a stationary solution to such equation. The stability of this homogeneous distribution is checked by adding a small perturbation to it, so that *ρ*(**r**, *t*) = *ρ*_0_ + *ϵψ*(**r**, *t*) (*ϵ* ≪ 1). Inserting this into Eq (4) we find that the perturbation growth rate of ψ∝exp(k·r+λt) is given by

$$\lambda \left(k\right)=-\frac{D_{0}}{2}(1+\tanh\gamma +\frac{2c\rho_{0}{\hat{G}}_{l}\left(k\right)-2b\rho_{0}{\hat{G}}_{s}\left(k\right)}{\cosh^{2}\gamma })k^{2},$$ (7)

where *γ* = 2(*a*−*bρ*_0_+*cρ*_0_)−1. ${\hat{G}}_{s}\left(k\right)$ and ${\hat{G}}_{l}\left(k\right)$ are the Fourier transforms of the short-range and the long-range kernels, respectively. Given the choice made for the kernels (Eq (6)), the Fourier transforms are

$${\hat{G}}_{\mu }\left(k\right)=2\frac{J_{1}\left(kR_{\mu }\right)}{\left|k\right|R_{\mu }},\;$$ (8)

where *μ* = *s* or *μ* = *l*, and *J*_1_ is the first order Bessel function. The homogeneous distribution is unstable and then patterns would appear if the maximum of the growth rate (i.e., of the most unstable mode), λ(**k**_*c*_), is positive, which means that the perturbation of periodicity 2*π*/|**k**_*c*_| grows with time. *λ* is showed for different values of the parameters *b* and *c* in Fig 5. Depending on the value of *b* and *c* the model shows two different types of instabilities. Instability A has stable low wavenumbers (green curve in Fig 5, see inset) that prevent the clusters to grow. The characteristic wavelength of the pattern is well defined around *k*_*c*_ = 49.52. On the other hand an instability of type B has a band of unstable modes starting at *k* = 0, which could allow the clusters to experience some coarsening in time. We observe that labyrinthic structures are formed by this type B instability.

**Fig 6:**
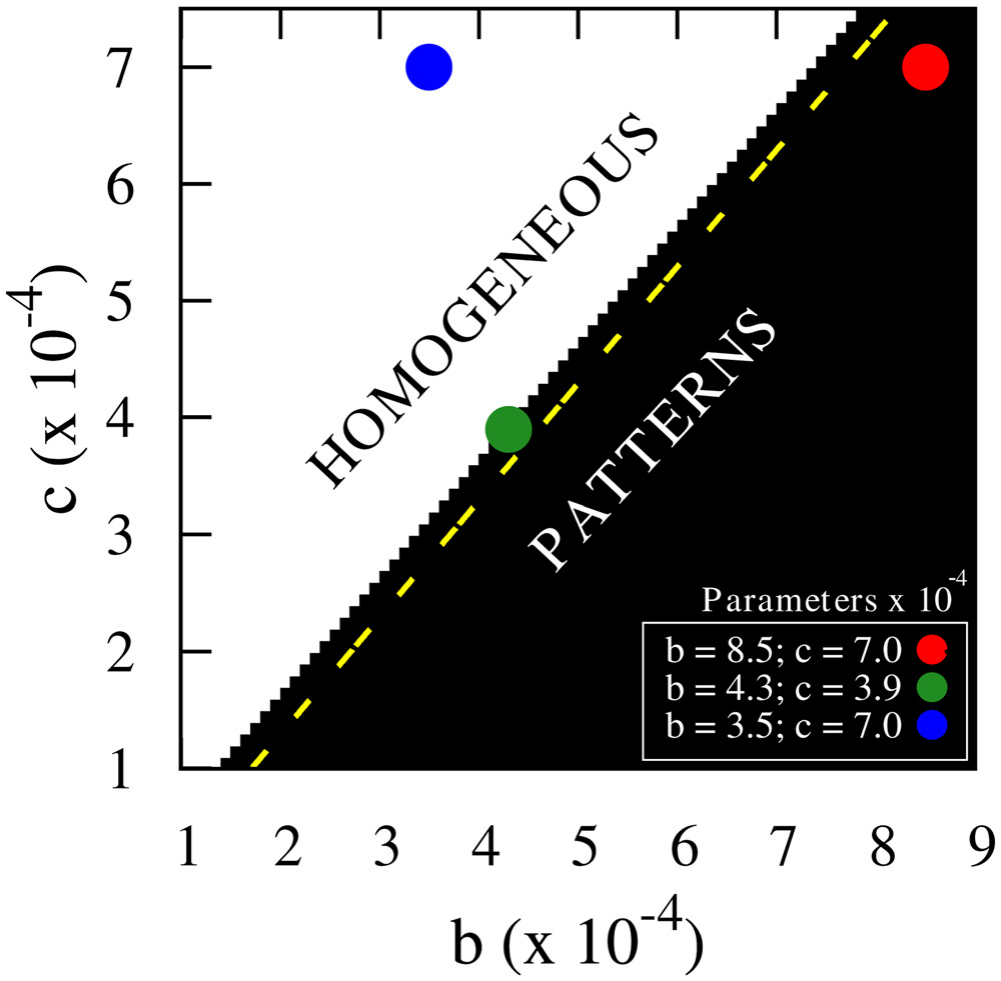
Phase diagram of the continuous approach. Parameter space of the continuous model where the regions of patterns and homogeneous solutions have been identified using the perturbation growth. *R*_*s*_ = 0.05, *R*_*l*_ = 0.1, *D*_0_ = 10^−4^ and *a* = 1. The yellow dashed line shows the transition from Instability A (above the line) to Instability B (below the line).

Evaluating the perturbation growth rate in Eq (7) with Eq (8), we may compute the phase diagram of the model (see Fig 6) for parameters *b* and *c* that gives information about the final spatial distribution of the system, homogeneous or patterned. The reduction of the diffusivity at high short-range densities is the responsible of the formation of patterns since clusters appear when *b* > *c*, that is when *ρ*_*s*_ is more relevant for the dynamics than *ρ*_*l*_. On the other hand, considering the effect of the long-range density alone on the diffusivity, the system shows homogenous distributions regardless of the value of *c* when *b* = 0. These are expected results since high values of the short-range density reduce the mobility of the individuals promoting clustering, while high values of the long-range density enhance longer displacements in the population, thus leading to homogenous distributions. However, the instability caused by a diffusivity reduction is enhanced by the presence of the *ρ*_*l*_ dependence because animals in-between two aggregations make longer displacements that allow them to reach a group. A similar argument has been used to explain the formation of clusters of species [44] and vegetation [45] in systems that only present long-range competitive interactions.

In addition, the boundary between both types of instabilities (A for hexagonal clusters, and B for labyrinthic patterns) is given by a change in the sign of the second derivative of the perturbation growth rate at *k* = 0. It is represented in Fig 6 by the yellow dashed line resulting from numerically evaluating

$$\lambda^{\prime \prime }{\left(k\right)|}_{k=0}=\frac{-D_{0}}{2}(1+\frac{2(c-b)}{\cosh^{2}\gamma }+\tanh\gamma )=0.$$ (9)

**Fig 7:**
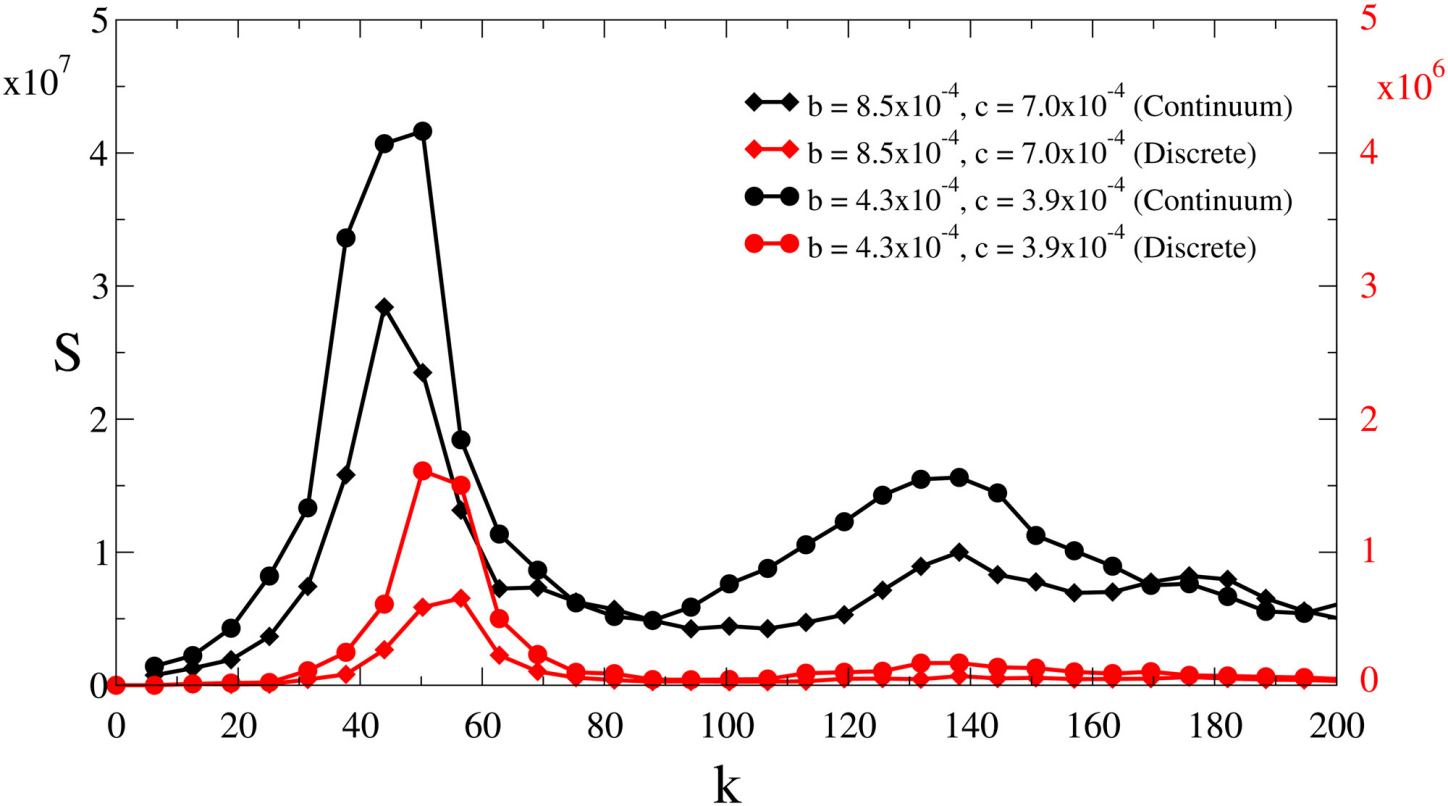
Structure functions. Structure function of the patterns obtained with the continuous and the discrete model for the case of labyrinthic and spotted patterns.

The typical scale of the pattern, that is, the distance between aggregates, can be studied with the structure function (Fig 7). It can be computed for both the patterns of particles and the density distribution. In the first case it is $S_{d}\left(k\right)=\langle {\left|\frac{1}{N}\sum_{j=1}^{N}e^{ik\cdot r_{j}}\right|}^{2}\rangle$, where **r**_*j*_ is the position vector of particle *j*, **k** is a two-dimensional wave vector with modulus *k*, and the average indicates a spherical average over the wave vectors with modulus *k* and in time. In the continuous approach, the structure function is calculated as the modulus of the spatial Fourier transform of density field, averaged spherically and in time. Note that both quantities are related but not identical, and their first maximum, *k*_*c*_, allows to compute the typical distance between clusters *d* = 2*π*/*k*_*c*_. For the spotted patterns *k*_*c*_ = 50.24 (discrete) and *k*_*c*_ = 49.52 (continuum) so that *d* ≈ 0.125–0.126. Regarding the case of the labyrinth pattern (central panel of Figs 2 and 3), *k*_*c*_ = 56.52 (discrete) and *k*_*c*_ = 51.31 (continuum), so that the typical distance between aggregates is *d* ≈ 0.11.

**Fig 8:**
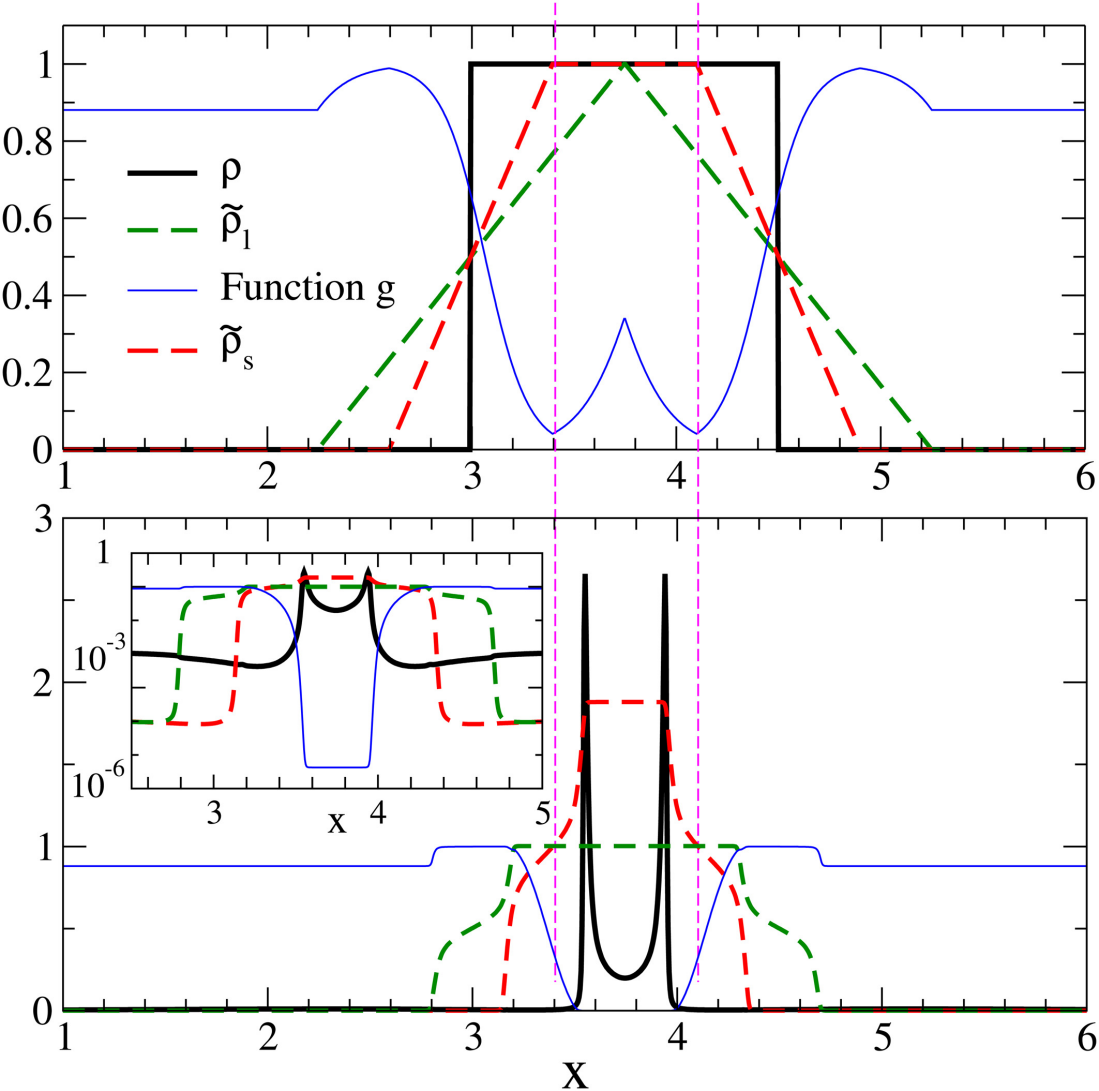
Formation of ring-like structures in the 1D model. Evolution of the 1D version of the model starting from an initial condition for *ρ*(*x*, *t* = 0) consisting on a pulse of height unity and length 2*R*_*l*_ (displayed in the upper panel). The legend indicates the quantity represented by the different lines. The two vertical lines indicate the minima of the function *g*(*x*) (i.e. the diffusion coefficient in units of *D*_0_) at *t* = 0 where particles will initially tend to accumulate. The bottom panel represent the same quantities (although *ρ*(*x*, *t*) has been divided by a factor 5 to fit into the same scale as the other curves) after a very long integration time (*t* = 6 × 10^5^). A double-peak structure has developed. The inset displays this long-time configuration in logarithmic scale, showing that *g*(*x*) ≈ 4 × 10^−6^ in the central region. Parameters: *a* = 1, *b* = 3.33, *c* = 2.67, *D*_0_ = 10^−4^, *R*_*l*_ = 0.75, *R*_*s*_ = 0.4, *N* = ∫ *dxρ*(*x*, *t* = 0) = 1.5

**Fig 9:**
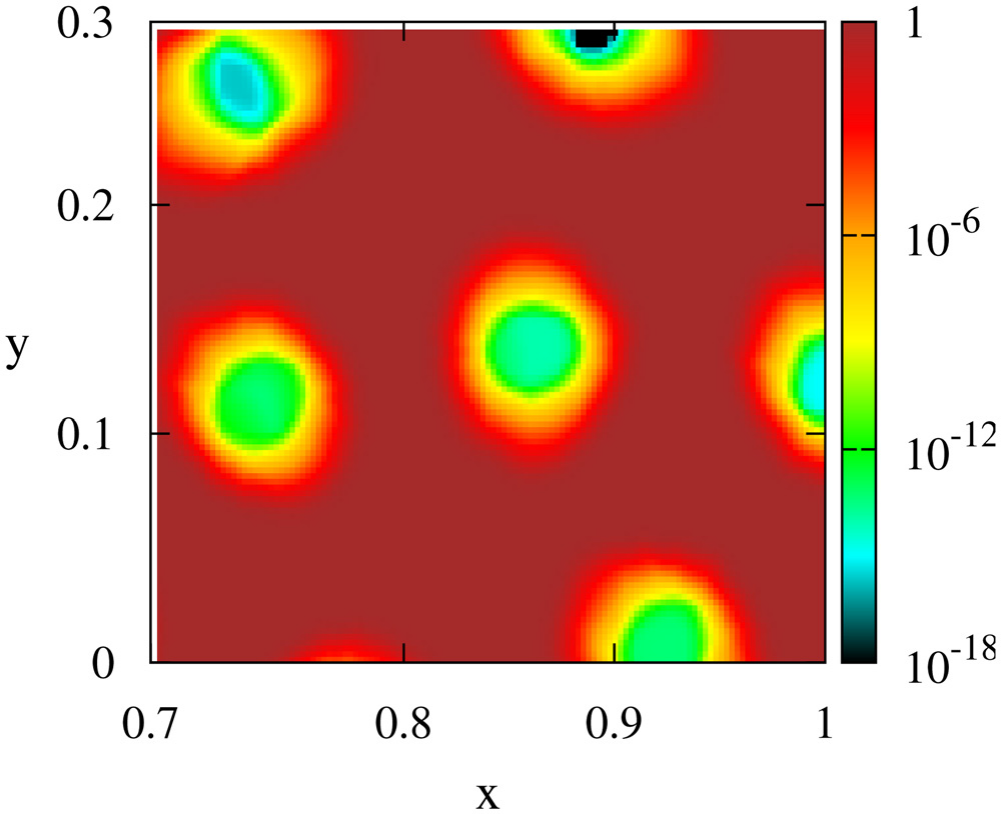
Diffusion field in the 2D model. Numerical computation of the function *g* as defined in Eq (3) from the spot pattern showed in Fig 3. *g* is extremely small inside the clusters, but not zero. Parameters: *R*_*s*_ = 0.05, *R*_*l*_ = 0.1, *D*_0_ = 10^−4^, *a* = 1, *ρ*_0_ = 10^4^, *b* = 4.3 × 10^−4^, *c* = 3.9 × 10^−4^. Zoom over the right lower corner of the pattern.

As it was stated before, the ring-like shape of the clusters deserves further consideration. To go deeper into this question we use the one-dimensional version of the model starting from an initial condition consisting of a single pulse of height unity (top panel of Fig 8). The mean nonlocal densities ${\tilde{\rho }}_{s}$ (dashed red line) and ${\tilde{\rho }}_{l}$ (dashed green line) can be easily obtained and lead to a diffusivity which in units of *D*_0_ is the function *g*, with two minima where particles will tend initially to clump (magenta vertical dashed lines in Fig 8). As time advances a two-peak distribution establishes, which is the one-dimensional analogue of the two-dimensional rings observed before. This double peak, of a spatial size close to *R*_*s*_, persists for extremely long times. However the inset in the bottom panel of Fig 8 shows that the diffusion coefficient in between the two peaks takes a nearly constant value which is very small but not zero (*g*(*x*) = *D*(*x*)/*D*_0_ ≈ 4 × 10^−6^). This implies that at still longer times (of the order of $R_{s}^{2}/D\approx 4\times 10^{5}$ after the time displayed in the bottom panel of Fig 8) particles will diffuse between the two peaks, replacing them by a homogeneous distribution. The same will occur in two dimensions, since as showed in Fig 9, the diffusion coefficient in the two-dimensional system is also homogeneous (but very small) inside the clusters so that at extremely long times the pattern of hollow clusters of Fig 3 will be replaced by homogeneous clusters. Thus the ring structures seem to be a very-long lived transient state. They will disappear faster if the prescription in Eq (3) for *g* is changed by another functional form with higher minimum values. Alternatively, for a choice such that *g*(*x*) is strictly zero for $\tilde{\rho_{s}}\gg \tilde{\rho_{l}}$ then the rings will persists for infinite time as stationary structures.

## Discussion

We have studied how the combination of a short-range inhibition and a long-range activation in individual dispersal may influence the long-time spatial distribution of a population, which ranges from homogeneous to labyrinth and spot patterns depending on the relative weights of each mechanism. This type of behavior has been observed in mussel beds [35–37] where individuals tend to clump at short distance as a defensive strategy while competition for resources acts at a larger scale.

Pattern formation arises as a consequence of the interplay between inhibition and activation acting at different spatial scales that makes the spatially homogeneous state to lose its stability. Resulting patterns show not only an inhomogeneous distribution of the population at a system level but also a non-uniform distribution of the individuals within each cluster. For the time scales discussed here ring-like structures are formed, with most of the particles at the borders of the groups. This point has been studied from a simplified 1D situation starting from an initial density given by a step function. Beyond the limits of the profile the nonlocal long-range densty is higher than the nonlocal short-range density. However, due to their different slopes, this situation is reversed and the short-range density becomes higher than the long-range one. This leads to the formation of annular structures. This mechanism will act for any kind of initial condition wherever there is a region where eventually the density is higher than in the rest of the system. Whether the rings will homogenize at very long times or rather they will remain stable depends on the details of the small-diffusion part of the density-dependent diffusivity.

The particular shape of the structures depends on the relative importance of the short and long-range mean densities, weighted by the values of the parameters *b* and *c*. The first is the responsible of the formation of aggregates, so the model gives homogeneous distributions when this scale tends to zero (*R*_*s*_ → 0 or equivalently *b* = 0). The larger one enhances the formation of groups. Individuals that do not belong to any group are surrounded by low densities in their close neighborhoods, but still can be in very far-populated regions. In these cases their movement has a larger diffusivity, so longer displacements are possible, increasing the probability of finding a group in a shorter time. A combination of both, a short- and a long-range dependence mobility, is an optimal mechanism to promote the formation of groups. In addition, the long-range competition stabilizes the ring-like structures since it avoids the formation of highly packed groups in a small area.

The generality of the model, a nonlinear diffusion equation with two nonlocal interaction scales that enhance and inhibit animal mobility, allows its application to a wide variety of ecological situations with these two ingredients. Moreover, our mathematical scheme shows a sequence of patterns that has been previously reported in natural systems such as mussel beds [36] (isolated spots spatially arranged at random can also be observed for slightly different setups of the model, for example, changing the hyperbolic tangent function in Eq (3)). In the case of mussels, long-range activation of dispersal arises from resources competition. Individuals would tend to escape from regions already colonized by other groups. Nevertheless they will remain inside a small group if at this smaller scales clustering provides some advantage such as protection against wave stress. Protection against predators is also a widespread benefit of clustering in groups. Within our approach, we recover spatial structures both in a stochastic and a deterministic description of the problem, suggesting that they are a result of the interplay between the two types of interactions with fluctuations playing a secondary role. Remarkably, our results bear similarities with results on vegetation patterns and fairy circles in arid regions [42, 43, 46] which arise from very different mechanisms, but have in common with our case the presence of competitive and facilitative interactions. We hope that our studies help the development of new mathematical models and more precise understanding of those situations where spatial distributions similar to the ones presented here are observed.
